# Associations of breast cancer etiologic factors with stromal microenvironment of primary invasive breast cancers in the Ghana Breast Health Study

**DOI:** 10.21203/rs.3.rs-2791342/v1

**Published:** 2023-04-14

**Authors:** Mustapha Abubakar, Thomas U. Ahearn, Maire A. Duggan, Scott Lawrence, Ernest Adjei, Joe-Nat Clegg-Lamptey, Joel Yarney, Beatrice Wiafe-Addai, Baffour Awuah, Seth Wiafe, Kofi Nyarko, Francis Aitpillah, Daniel Ansong, Stephen M. Hewitt, Louise A. Brinton, Jonine D. Figueroa, Montserrat Garcia-Closas, Lawrence Edusei, Nicolas Titiloye

**Affiliations:** National Cancer Institute; National Cancer Institute; University of Calgary; Leidos Biomedical Research, Inc; Komfo Anokye Teaching Hospital; Korle Bu Teaching Hospital; Korle Bu Teaching Hospital; Peace and Love Hospital; Komfo Anokye Teaching Hospital; Loma Linda University; University of Ghana; Komfo Anokye Teaching Hospital; Kwame Nkrumah University of Science and Technology; National Cancer Institute; National Cancer Institute; University of Edinburgh; National Cancer Institute; Korle Bu Teaching Hospital; Komfo Anokye Teaching Hospital

## Abstract

**Background:**

Emerging data suggest that beyond the neoplastic parenchyma, the stromal microenvironment (SME) impacts tumor biology, including aggressiveness, metastatic potential, and response to treatment. However, the epidemiological determinants of SME biology remain poorly understood, more so among women of African ancestry who are disproportionately affected by aggressive breast cancer phenotypes.

**Methods:**

Within the Ghana Breast Health Study, a population-based case-control study in Ghana, we applied high-accuracy machine-learning algorithms to characterize biologically-relevant SME phenotypes, including tumor-stroma ratio (TSR (%); a metric of connective tissue stroma to tumor ratio) and tumor-associated stromal cellular density (Ta-SCD (%); a tissue biomarker that is reminiscent of chronic inflammation and wound repair response in breast cancer), on digitized H&E-stained sections from 792 breast cancer patients aged 17–84 years. Kruskal-Wallis tests and multivariable linear regression models were used to test associations between established breast cancer risk factors, tumor characteristics, and SME phenotypes.

**Results:**

Decreasing TSR and increasing Ta-SCD were strongly associated with aggressive, mostly high grade tumors (*p-value* < 0.001). Several etiologic factors were associated with Ta-SCD, but not TSR. Compared with nulliparous women [mean (standard deviation) = 28.9% (7.1%)], parous women [mean (standard deviation) = 31.3% (7.6%)] had statistically significantly higher levels of Ta-SCD (*p-value* = 0.01). Similarly, women with a positive family history of breast cancer [FHBC; mean (standard deviation) = 33.0% (7.5%)] had higher levels of Ta-SCD than those with no FHBC [mean (standard deviation) = 30.9% (7.6%); *p-value* = 0.01]. Conversely, increasing body size was associated with decreasing Ta-SCD [mean (standard deviation) = 32.0% (7.4%), 31.3% (7.3%), and 29.0% (8.0%) for slight, moderate, and large body sizes, respectively, *p-value* = 0.005]. These associations persisted and remained statistically significantly associated with Ta-SCD in mutually-adjusted multivariable linear regression models (*p-value* < 0.05). With the exception of body size, which was differentially associated with Ta-SCD by grade levels (*p-heterogeneity* = 0.04), associations between risk factors and Ta-SCD were not modified by tumor characteristics.

**Conclusions:**

Our findings raise the possibility that epidemiological factors may act via the SME to impact both risk and biology of breast cancers in this population, underscoring the need for more population-based research into the role of SME in multi-state breast carcinogenesis.

## Introduction

In addition to the neoplastic parenchymal cells, tumors are comprised of a complex mixture of cellular and non-cellular (extracellular matrix (ECM)) elements that collectively comprise the stromal microenvironment (SME) [1–4]. The SME plays a critical role in tumor initiation, progression, and response to treatment [1, 5–8]. Although widely believed to evolve in parallel with the neoplastic parenchyma, accumulating data suggest that changes in the SME may precede breast cancer development and that premalignant/pre-invasive SME phenotype could influence the biologic phenotype of ensuing tumors [9–11].

Emerging lines of evidence suggest that host factors, such as parity, body mass index (BMI), and race/ethnicity may impact the SME [12–15]. This is of particular relevance given the observed heterogeneity in the incidence of breast cancer subtypes according to host factors [16, 17]. For instance, parity has been shown in several epidemiological studies to be more strongly associated with risks of triple negative breast cancer (TNBC), an aggressive form of breast cancer that is characterized by lack of expression of estrogen receptors (ER), progesterone receptors (PR), and human epidermal growth factor receptor 2 (HER2)), while postmenopausal obesity has been shown to more strongly predispose to risks of high grade/ER + tumors [18–21]. Other risk factors, such as early menarche, nulliparity, lack of breastfeeding, postmenopausal status, and use of menopausal hormone therapy (MHT) have been shown to be more consistently associated with elevated risks of ER + than ER− breast cancer subtypes [16, 18, 22]. Furthermore, compared with women of European ancestry, women of African ancestry tend to have a higher prevalence of aggressive, TNBC or high grade, breast cancer phenotypes [23–25]. Similar patterns of higher rates of aggressive breast cancer phenotypes are seen among women in Sub-Saharan Africa [26, 27].

Although most previous studies of breast cancer etiologic heterogeneity have focused on tumor parenchymal characteristics such as ER, PR, and HER2, distinct SME phenotypes may contribute to observed differences in tumor biology by host factors [12, 13, 15, 28]. In general, SME phenotypes reminiscent of chronic inflammation and wound repair have been shown to predominate among parous than nulliparous, obese than normal weight, and African American women [15, 28]. In particular, and with respect to the latter, the densities of tumor-associated macrophages, endothelial cells, and micro-vessels in the SME, in addition to the expression of an interferon signature, have been shown to be higher among women of African than European ancestry after accounting for age, tumor stage, ER subtype, and grade [15]. To date, however, there have been no studies investigating the impact of host on the SME of breast cancer among women from an indigenous sub-Saharan African population who are more likely to develop aggressive breast cancer subtypes and less likely to undergo screening.

Our main aim in this study was to investigate the association between host factors and SME phenotypes among women with breast cancer from a sub-Saharan African population. To address this aim, we utilized high-accuracy machine learning algorithms to characterize the SME of primary invasive breast cancer using digitized hematoxylin and eosin (H&E)-stained sections of tumor tissues. Two biologically-relevant H&E-based SME phenotypes were considered, including the tumor-stroma ratio (TSR), which is a metric of the proportion of tumor tissue that is stroma relative to the neoplastic parenchyma [29, 30]; and the tumor-associated stromal cellular density (Ta-SCD), which is a metric of the proportion of the tumor-stroma that is occupied by nucleated cells such as immune cells (e.g., tumor infiltrating lymphocytes (TILs), macrophages, and natural killer (NK) cells), other cellular components such as fibroblasts, endothelial cells, and pericytes [31]. Relationships between breast cancer risk factors and SME phenotypes were assessed overall, and according to relevant clinicopathologic characteristics, including age, ER status, and histologic grade.

## Materials And Methods

### Study population

The Ghana Breast Health Study (GBHS) is a multidisciplinary, population-based, case-control study in Ghana, details of which have been previously described [32]. In brief, cases were women who presented with lesions suspicious for breast cancer in three hospitals in Kumasi (Komfo Anokye Teaching Hospital and Peace and Love Hospital) and Accra (Korle Bu Teaching Hospital), Ghana, between 2013 and 2015. These hospitals represent the primary hospitals that provide surgical and oncological care for breast cancer patients in Ghana [32]. A total of 1,126 breast cancer patients were recruited as part of the GBHS [33]. Of these, 792 had available digitized H&E-stained images that were confirmed by study pathologists to contain invasive breast cancer. Accordingly, the current analysis is comprised of 792 patients aged 17–84 years with histologically confirmed invasive breast cancer and for whom we had digitized H&E-stained images. The study was approved by institutional review boards at the National Institutes of Health (NIH; Institutional Review Board of the National Cancer Institute), Kwame Nkrumah University of Science and Technology (Kumasi, Ghana), Noguchi Memorial Institute for Medical Research (Accra, Ghana), School of Medical Sciences, Komfo Anokye Teaching Hospital (Kumasi, Ghana) and Westat (Rockville, MD, USA). All participants provided written informed consent.

### Risk factor information

Data on established breast cancer risk factors, including demographic factors, menstrual and reproductive characteristics, family history of breast cancer (FHBC), medical history, occupational history, anthropometric, and physical activity variables, were obtained using a detailed questionnaire that was administered to each participant in the hospital by trained personnel. The women were asked specifically about pregnancy and breastfeeding practices, including number of child births and total duration of breastfeeding for each birth. Data on average lifetime body size were obtained using pictograms that were classified as slight, average, slightly heavy, and heavy [32]. This study relied on pictograms due to lack of scales in some places and to overcome the challenge of weight loss due to disease. For this analysis, we used risk factor variables that have been previously described and shown to be associated with breast cancer risk in this population [32, 33]. Participants’ ages (years) were categorized as < 35, 35–44, 45–54, and ≥ 55 years. Age at menarche (years) was categorized as < 15, 15, 16, ≥ 17. Parity was classified as nulliparous, 1–2, 3–4, and ≥ 5 children. Age at first birth (years) was categorized as < 19, 19–21, 22–25, and ≥ 26. Breastfeeding (median, per birth, months) was categorized as < 13, 13–18, and ≥ 19. Body size phenotypes were classified as slight, moderate, or heavy. FHBC was considered as positive if a patient had a history of breast cancer in a first degree relative and negative if there was no such history.

### Tumor pathology data

Pre-treatment core-needle biopsies (n = 4–8) from each patient were fixed in 10% neutral buffered formalin for 24–72 hours and subsequently processed into paraffin-embedded tissue blocks [32]. Data on tumor size were based on clinical palpation of the mass at the time of diagnosis [34]. Information on histologic grade was obtained through centralized pathologist (MAD) review [35]. Immunohistochemical (IHC) staining for ER, PR, HER2 were performed using standard laboratory protocols [33]. Tumors were considered to be ER + and/or PR + if ≥ 10% of the tumor cells demonstrated positive staining. 3 + staining for HER2 was considered as positive while borderline and negative cases were considered as HER2 negative. As previously reported [33], good agreements (79%, 65%, and 78% for ER, PR, and HER2, respectively; P < 0.01) were observed between scores that were determined by pathologists in Ghana and at the NCI. Breast cancer subtypes were defined using data on ER, PR, and HER2 as follows: luminal A-like (ER+/PR+/HER2−); luminal B-like (ER + and/or PR+/HER2 + or ER+/PR−/HER2−); HER2-enriched (ER−/PR−/HER2+) and TNBC (ER−/PR−/HER2−).

### Machine learning characterization of stromal microenvironment using hematoxylin and eosin (H&E) stained images

H&E-stained sections were digitized at the NCI and archived using the Halo Link digital image repository (Indica Labs, Albuquerque, NM). Imaging quality control (QC) was performed by pathologists (NT, LE, MA) using a standard operating protocol designed to identify and positively annotate tumor regions on the slide (including intra-tumoral and peri-tumoral stroma), and to negatively annotate regions with substantial crushing artifacts, damaged tissue, or widespread necrosis. In addition, pathologists used a computer-assisted visual counting tool to identify and count nucleated cells within well-defined regions (500×500μm^2^) of stroma.

**Figure 1:**
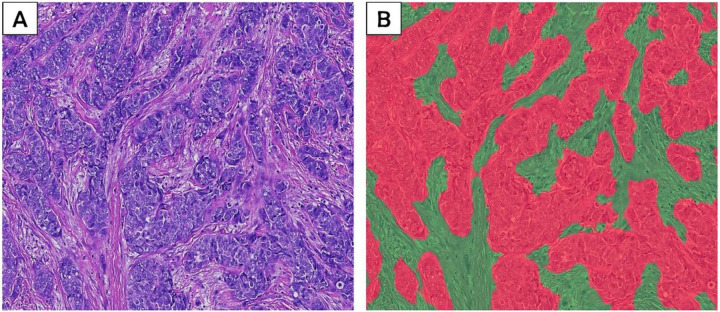
Machine-learning classification of stromal and tumor tissue compartments.

**Figure 2:**
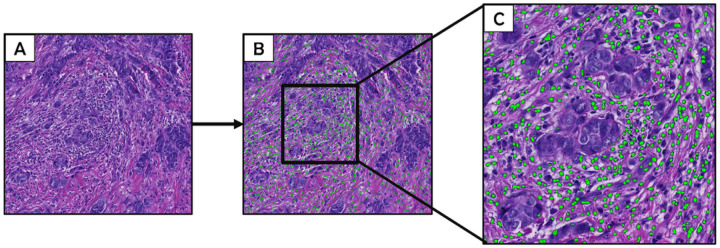
Machine-learning classification of tumor-associated stromal cellular density.

Digitized images were analyzed using optimized machine-learning scripts based on the random forest algorithm. First, a 160-datapoint random forest tissue-classifier script was trained and optimized to identify, segment, and quantify (in mm^2^) areas on each image consisting of tumor (94-datapoints) and stroma (67-datapoints) (Fig. 1). The performance of the tissue classifier script in distinguishing between tumor and stroma was confirmed by visual inspection of random images by study pathologists. As previously reported in an independent study population [36], the random forest tissue-classifier demonstrates excellent reproducibility when comparing scripts that were independently trained by two pathologists (Spearman’s rho = 0.95 and 0.97 for epithelium and stroma, respectively) [36]. Stromal regions were digitally annotated to allow subsequent cell detection to be confined to the stromal compartment. Next, a previously validated [31] cell-detection script was reparametrized (**Additional file 1**) to identify and count nucleated cells in the stroma including lymphocytes, macrophages, fibroblasts, endothelial cells, etc. (Fig. 2). In validation analysis, the cell detection script showed strong correlation with two pathologists’ manual cell counts within the intra-tumoral (R = 0.93 and 0.75 *vs* P1 and P2, respectively) and peri-tumoral (R = 0.74 and 0.73 *vs* P1 and P2, respectively) stromal compartments. Optimized scripts were used for centralized image analysis, which was blinded to patient’s demographic, epidemiological, or clinical characteristics.

Percent TSR was calculated by dividing the stromal area (mm^2^) by total fibroglandular tissue area on the slide and multiplying by 100. Ta-SCD was calculated by dividing the total number of cells in the stroma by the total stromal area (mm^2^). This was converted to a percentage by multiplying the total number of nucleated cells by the average area (mm^2^) of a single nucleus (2.0×10^− 04^), dividing this by the total stromal area (mm^2^), and multiplying by 100. There was near-perfect positive correlation (r = 0.99; **Additional file 2**) between standard and percent Ta-SCD. The latter was used for all further analysis to facilitate interpretability.

Digitized hematoxylin and eosin (H&E)-stained images (**A**) were analyzed using optimized machine-learning scripts based on the random forest algorithm. A 160-datapoint random forest tissue-classifier was trained and optimized to identify, segment, and quantify (in mm^2^) areas on H&E image consisting of tumor (94-datapoints, red regions (**B**) and stroma (67-datapoints; green regions (**B**)).

Stromal regions on digitized hematoxylin and eosin (H&E)-stained images (**A**) were digitally annotated to allow subsequent cell detection to be confined to the stromal compartment. The cell detection script was parameterized to detect non-malignant nucleated cells in the stroma (green dots; B and C) based on size, shape, nuclear detection weight, nuclear contrast threshold, and optical density (Additional file 1). The algorithm was trained to exclude infiltrating nests of malignant epithelial cells from the tumor-associated stromal cellular density metric (C). The cell detection script showed strong correlation with two pathologists’ manual cell counts within the intra-tumoral (R = 0.93 and 0.75 vs P1 and P2, respectively) and peri-tumoral (R = 0.74 and 0.73 vs P1 and P2, respectively) stromal compartments.

### Statistical analysis

Kruskal Wallis test was used to evaluate differences in the distributions of TSR and Ta-SCD by patients’ characteristics. Multivariable linear regression models were used to test associations between risk factors, patients’ clinicopathological characteristics, and SME characteristics. TSR and Ta-SCD were normally distributed hence generalized linear regression was used to test associations with risk factors and tumor characteristics. In separate linear regression models, TSR and Ta-SCD were modelled as outcomes while the risk factors and patients’ characteristics were modelled as predictors. Partially-adjusted models contained the relevant risk factor in addition to age, study site, and tissue area. The multivariable model was mutually adjusted for the individual risk/clinicopathological factors in addition to age, study site and tissue area. Multiplicative interaction terms were included in full models to test for evidence effect modification between host factors and tumor characteristics in relation to the SME characteristics. Missing covariate values on tumor characteristics were imputed using the multiple (×5) imputation by chained equations (MICE) approach [37] with appropriate variance adjustment by Rubin’s Formula [38] for all analyses. All analyses were two-sided and were performed using Stata statistical software version 16.1. P values < 0.05 were considered statistically significant.

## Results

### Description of patient characteristics and distributions of stromal microenvironment features by tumor characteristics

This analysis comprised 792 patients aged 17–84 (mean = 49.8, median = 49.0) years at diagnosis. 52% of the patients had ER + and/or PR + tumors, while 24% had HER2 + disease. The majority (~ 70%) of the patients had high (grade 3) disease and about 6% had low (grade 1) grade tumors. 97% of the tumors in this population were at least 2cm in size at diagnosis. The distributions of breast cancer subtypes were 30%, 34%, 8%, and 28% for luminal A-like, luminal B-like, HER2-enriched, and TNBC, respectively.

The median age at menarche was 15 (range = 9–22) years. About 91% of the population had at least one child at the time of breast cancer diagnosis, with the majority having 3 or more children. 32% of the population breastfed for < 13 months/pregnancy while 13% breastfed for at least 19 months/birth. The distributions of body size phenotypes were 25%, 40%, and 35% for slight, moderate, and heavy. 55 patients (~ 7%) reported having a positive FHBC (Table 1).

The mean, median (range) of TSR (%) and Ta-SCD (%) were 76.2, 78.9 (13.9–99.6) and 31.0, 30.1 (10.7–58.7), respectively (Table 2). Of the clinicopathological factors, the distributions TSR and Ta-SCD differed significantly by histologic grade. In general, TSR levels decreased with increasing grade (P < 0.0001). Conversely, Ta-SCD increased with increasing grade levels (P < 0.0001). We did not find the distribution of TSR or Ta-SCD levels to differ according to other clinicopathologic characteristics (Table 2).

### Associations between breast cancer clinicopathological and molecular characteristics and stromal microenvironment features

In linear regression models partially adjusted for individual tumor characteristic as well as age, hospital site, and tissue area, we found ER, HER2, TNBC, and histologic grade to be statistically significantly associated with TSR. Following mutual adjustments for all tumor characteristics, ER [β (95% CI) _ER+ vs ER−_ = 2.91 (0.19, 5.63); P = 0.03], HER2 [β (95% CI) _HER2+ vs HER2−_ = 4.39 (1.43, 7.35); P = 0.005], and histologic grade [β (95% CI) = −2.07 (−6.70, 2.56), −9.19 (−13.40, −4.98) for grades 2 and 3 *vs* 1, respectively; P-trend < 0.001] remained statistically significantly associated with TSR (Table 3). Ta-SCD was statistically significantly higher with increasing grade in partially-adjusted models and after adjustment for other clinicopathological factors in multivariable models [β (95% CI) = 3.53 (1.03, 6.03), 5.22 (2.74, 7.71) for grades 2 and 3 *vs* 1, respectively; P-trend < 0.001]. Increasing Ta-SCD was also suggestively associated with increasing tumor size, but this did not attain statistical significance [β (95% CI) = 0.07 (−3.10, 3.23), 1.20 (−1.95, 4.36) for 2–5 and > 5 vs < 2cm, respectively; P-trend = 0.06] (Table 3).

### Associations between breast cancer etiologic factors and stromal microenvironment features

The distributions of Ta-SCD (Table 2), but not TSR, varied statistically significantly according to categories of parity, body size, and FHBC. Ta-SCD was higher among parous [mean (standard deviation) = 31.3% (7.6%)] than nulliparous [mean (standard deviation) = 28.9% (7.1%)] women (P-value = 0.01) and was higher among women with a positive [mean (standard deviation) = 33.0% (7.5%)] than those with no [mean (standard deviation) = 30.9% (7.6%)] FHBC (p-value = 0.01). Conversely, increasing body size was associated with decreasing Ta-SCD [mean (standard deviation) = 32.0% (7.4%), 31.3% (7.3%), and 29.0% (8.0%) for slight, moderate, and large body sizes, respectively, p-value = 0.005]. These findings persisted and remained statistically significant in mutually-adjusted multivariable linear regression models (Table 4). Specifically, parity [β (95% CI) vs nulliparous = 2.92 (1.04, 4.81); p-value = 0.002] was associated with higher Ta-SCD after accounting for the other risk factors, but there was no trend with increasing number of births (Table 4). There were no associations between Ta-SCD and age at first birth (AFB) or breastfeeding (BF). Further, BF duration did not appear to attenuate the association between parity and Ta-SCD [β (95% CI) _parous/BF vs nulliparous_ = 2.48 (0.37, 4.48), p-value = 0.02; 3.34 (1.39, 5.30), p-value = 0.001; and 3.36 (0.93, 5.78), p-value = 0.007 for parous/BF < 13months, parous/BF ~ 13–18months, parous/BF ≥ 19months, respectively]. Having a positive FHBC [β (95% CI) _vs no FHBC_ =2.36(0.28, 4.44), p-value = 0.02] was also statistically significantly associated with higher Ta-SCD, while increasing body size was statistically significantly inversely associated with Ta-SCD [β (95% CI) = −1.02 (−2.42, 0.39), −2.42 (−3.84, −1.01) for moderate and heavy *vs* slight body size, respectively; p-trend = 0.001] (Table 4). No risk factor was statistically significantly associated with TSR (Table 4).

We did not find evidence to suggest modification of the associations between parity, parity/BF, or family history and Ta-SCD by age (< 50 vs ≥ 50 years; **Additional file** 3), ER status (ER + vs ER−; **Additional file 4**), or histologic grade (**Additional file 5**). Similarly, the association between body size and Ta-SCD did not differ according to age or ER status. The directionality of the association between body size and Ta-SCD, however, differed according to levels of histologic grade (p-value for heterogeneity = 0.04). Among patients with low grade tumors, increasing body size was positively associated with increasing Ta-SCD [β (95% CI) = 5.41 (−3.70, 14.52) and 6.20 (−3.89, 16.30) for moderate and heavy *vs* slight body size, respectively; P-trend = 0.18] while among those with intermediate/high grade tumors, increasing body size was inversely associated with decreasing Ta-SCD [β (95% CI) = −1.04 (−2.67, 0.59) and − 2.62 (−4.22, −1.03) for moderate and heavy *vs* slight body size, respectively; P-trend = 0.001] (**Additional file 5**).

## Discussion

In analyses of 792 breast cancer patients from the GBHS, a population-based case-control study in Ghana, we utilized high-accuracy machine learning algorithms to characterize H&E-based SME phenotypes, including TSR and Ta-SCD, and investigated relationships with breast cancer etiologic and tumor factors. We found TSR and Ta-SCD to be associated with tumor characteristics, particularly histologic grade. Several risk factors were associated with Ta-SCD, but not TSR. In particular, parity, FHBC, and body size were statistically significantly associated with Ta-SCD, independently of relevant tumor characteristics. The observed associations between individual risk factors and Ta-SCD were mostly consistent with their breast cancer risk relationships, suggesting that SME phenotype may reflect cumulative exposure to etiologic factors.

To date, theories of breast carcinogenesis have focused largely on sequences of epithelial abnormalities in tissues [5], but accumulating data suggest that the SME might play a crucial role in tumor initiation as well as tumor biology [1, 39]. For instance, chronic inflammation has been shown in experimental studies to contribute to tumor initiation by inducing malignant transformation of epithelial cells via inactivating mutations in tumor suppressor genes or through the posttranslational modification of proteins involved in apoptosis and DNA repair [10, 40]. In addition to experimental evidence, the potential role of SME in breast cancer initiation is supported by data from population-based studies suggesting that factors that decrease chronic inflammation, such as Aspirin and other non-steroidal anti-inflammatory drugs (NSAIDS), also reduce breast cancer risk [41–44]. Comprising nucleated stromal cells, including lymphocytes, macrophages, fibroblasts, endothelial cells, etc., Ta-SCD is reflective of morphological changes that are reminiscent of chronic inflammation and wound healing response [31]. Accordingly, our findings of strong associations between FHBC, parity, and body size with Ta-SCD, which were consistent with their breast cancer risk associations support the idea that epidemiological exposures may impact breast cancer risk by acting on the SME.

Our finding of higher Ta-SCD among parous than nulliparous women is consistent with experimental data showing that parity might influence breast tumor biology by inducing SME changes [45, 46]. In particular, genes associated with immune, inflammation, and wound response pathways have consistently been shown to be significantly upregulated among parous than nulliparous women [14, 47]. Together with our finding of associations between increasing Ta-SCD and aggressive, high grade, breast tumors in this and other populations [31], our results add to the growing body of literature indicating that parity might predispose to the development of aggressive breast cancer phenotypes through SME-related pathways [45]. This notion is particularly relevant among women in Ghana [33] and other sub-Saharan African populations who tend to have higher parity rates in parallel with higher incidence rates of aggressive, early-onset, breast cancer [25, 27]. Although breastfeeding is thought to attenuate parity-related risk of aggressive breast cancer [20], we found the association between parity and Ta-SCD to persist irrespective of breastfeeding duration. This finding could be explained in light of studies showing that post-lactational changes in mammary tissue, such as upregulation of genes related to immune response and development, do not revert to nulliparous levels several years after lactation [47].

Our findings of higher Ta-SCD levels in relation to positive FHBC and, to a lesser degree, earlier age at menarche are consistent with their well-established associations with elevated breast cancer risk [48]. The precise mechanisms by which a positive family history of breast cancer increases breast cancer risk is yet to be fully defined but is closely linked to a higher probability of carrying pathogenic variants in breast cancer predisposition genes. Mutations in breast cancer predisposition genes such as BRCA1, BRAC2, and TP53, lead to uncontrolled proliferation via perturbations in DNA repair mechanisms or loss of cell cycle control [49, 50]. Because these perturbations are not limited to epithelial cells, our SME-related findings might suggest that the associations between FHBC and breast cancer incidence and/or tumor biology may be mediated, at least in part, through SME disruption. Recent data from a large-scale international study showed that protein-truncating variants and pathogenic missense mutations in BRCA1, RAD51C, RAD51D, and BARD1 more strongly predisposed to TNBC than other breast cancer subtypes [51]. However, this study was mostly comprised of European ancestry populations and the mechanism by which genetic perturbations influenced TNBC incidence was not studied. Data from other studies showed that differences in ancestry-specific immunologic landscapes correlated with differences in TNBC biology and clinical outcomes [52]. Our data are consistent with the notion that SME biology (which encompasses immunologic response) might be a pathway by which familial factors may impact breast cancer incidence and tumor biology. Further studies are needed to characterize the relationships between specific germline pathogenetic variants and tumor SME phenotype among women from sub-Saharan African populations.

We found larger body size to be inversely associated with Ta-SCD. As a chronic inflammatory state [53], it is not clear why higher body size was associated with lower Ta-SCD in this population. A possible explanation may have to do with the complex relationship between BMI and tumor biology with respect to age, menopause, and immune response. For instance, higher BMI is more strongly associated with ER− tumors among younger/premenopausal women [54] and with ER + tumors among older/postmenopausal women [55]. However, within ER + tumors, higher BMI is reportedly associated with more aggressive high-grade subtypes [21]. Accordingly, our observed inverse association between body size and Ta-SCD in the overall analysis may be reflective of the predominant tumor pathology in this population. ~95% of the tumors in this study were intermediate- (~ 25%) or high- (~ 70%) grade and the inverse association between increasing body size and decreasing Ta-SCD was limited to these aggressive tumor subtypes. However, and in keeping with the notion that higher body size may be a chronic inflammatory state, we found increasing body size to be positively associated with higher Ta-SCD among patients with low-grade disease. These findings could also be explained by the well-recognized paradoxical effect of obesity to cause suppression or activation of inflammatory response pathways in breast cancer [56, 57]. Immune exhaustion is another plausible explanation for the inverse association between larger body size and Ta-SCD [58]. T-cells mediate several aspects of tumor-associated inflammation and immune response, but they are themselves regulated by a complex crosstalk involving cancer cells, inflammatory cells, stromal cells, and cytokines. Persistent inflammation can overwhelm the T-cell response and trigger their terminal differentiation, leading to the emergence of ‘exhausted’ T-cell phenotypes with diminished capacity to mount immune or inflammatory responses [58]. Although speculative, the combination of obesity, a proinflammatory state, and prolonged and episodic exposure to malaria may predispose to immune exhaustion among women in sub-Saharan Africa. Immune exhaustion may, in turn, pave way for the development of more aggressive breast cancer subtypes via immunoediting [59]. Further studies involving detailed exposure assessment, including past medical and infectious disease history, in conjunction with detailed molecular characterization of the SME, will be required to lend additional insights into the relationships between body size and SME biology among women from sub-Saharan Africa.

Important strengths of this study include the relatively large sample size in an underrepresented population, detailed risk factor information, availability of standard H&E-stained sections, and the innovative application of digital pathology and high-accuracy machine learning algorithms to characterize SME phenotypes (TSR and Ta-SCD) using H&E-stained images. In terms of limitations, lack of follow-up data on clinical outcomes precluded our ability to investigate the prognostic relevance of TSR and Ta-SCD. Nevertheless, these SME features demonstrated associations with other clinicopathologic characteristics similar to what has been reported in other populations [29, 31], suggesting that they may be similarly associated with clinical outcomes among patients from Sub-Saharan Africa.

In conclusion, results from our study indicate that factors that impact the incidence of breast cancer in the general population might do so by influencing the SME in breast tissues. In particular, parity, FHBC, and body size demonstrated associations with stromal cellularity, captured using Ta-SCD. Notably, the observed associations between risk factors and Ta-SCD were consistent with their associations with breast cancer risk and/or tumor heterogeneity. Thus, these findings raise the possibility that some breast cancer etiologic factors may act via the SME to impact both risk and molecular phenotype of breast cancer.

## Figures and Tables

**Table 1:** Description of clinicopathologic and risk factor characteristics of 792 breast cancer patients participating in the Ghana Breast Health Study

Characteristic	Frequency (%)
Overall	792
Age, years	
< 35	84 (10.6)
35–44	198 (25.1)
45–54	231 (29.2)
≥ 55	277 (35.1)
Unknown	2
ER status	
Negative	287 (48.3)
Positive	307 (51.7)
Unknown	198
PR status	
Negative	282 (47.7)
Positive	309 (52.3)
Unknown	201
HER2 status	
Negative	422 (75.5)
Positive	137 (24.5)
Unknown	233
Grade	
Low	38 (5.6)
Intermediate	168 (24.7)
High	474 (69.7)
Unknown	112
Size	
< 2cm	23 (3.0)
2–5cm	248 (32.9)
> 5cm	483 (64.1)
Unknown	38
Subtype	
Luminal A-like	166 (30.0)
Luminal B-like	188 (33.9)
HER2-enriched	47 (8.4)
TNBC	154 (27.7)
Unknown	237
Age at Menarche, years	
<15	185 (27.0)
15	183 (26.7)
16	151 (22.0)
≥17	167 (24.3)
Unknown	106
Parity	
Nulliparous	71 (9.0)
Parous	719 (91.0)
Unknown	2
Number of children	
1	88 (12.2)
2	125 (17.4)
3	136 (18.9)
4	123 (17.1)
≥5	247 (34.4)
Age at first birth (years)	
<19	230 (30.8)
19–21	200 (26.8)
22–25	180 (24.1)
>26	137 (18.3)
Breastfeeding, months	
<13	240 (31.9)
13–18	415 (55.2)
≥19	97 (12.9)
Unknown	40
Joint parity/breastfeeding (months)	
Nulliparous	71 (9.4)
Parous/<13	169 (22.5)
Parous/13–18	415 (55.2)
Parous/≥19	97 (12.9)
Body Size	
Slight	190 (25.5)
Moderate	296 (39.7)
Heavy	260 (34.8)
Unknown	46
Family history	
None	726 (93.0)
Yes	55 (7.0)
Unknown	11

**Table 2:** Distributions of tumor microenvironment phenotypes (Tumor-stroma Ratio (TSR) and Tumor-associated Stromal Cellular Density (Ta-SCD)) by patients’ clinicopathological and risk factor characteristics in the Ghana Breast Health Study

	Tumor-Stroma Ratio (TSR)			Tumor-associated Stromal Cellular Density (SCD)		
Characteristic	Mean (SD)	Median (range)	*P value*	Mean (SD)	Median (range)	*P value*
Overall	76.2 (14.4)	78.9 (13.9–99.6)		31.0 (7.61)	30.1 (10.7–58.7)	
Age, years						
<35	77.6 (13.4)	77.7 (42.9–99.6)		31.6 (7.4)	31.4 (12.1–51.9)	
35–44	76.1 (16.4)	79.9 (13.9–98.8)		30.7 (7.7)	30.1 (13.0–54.3)	
45–54	75.7 (14.0)	77.9 (17.3–99.3)		30.3 (7.5)	30.4 (10.7–53.4)	
≥55	76.3 (13.9)	79.3 (21.8–99.1)	0.48	31.7 (7.6)	31.6 (12.4–58.7)	0.44
ER status						
Negative	75.1 (15.6)	78.0 (17.3–99.8)		31.1 (7.2)	30.7 (12.1–49.7)	
Positive	77.5 (12.7)	80.1 (17.9–98.9)	0.22	30.4 (7.7)	30.0 (10.7–58.7)	0.37
PR status						
Negative	76.9 (14.4)	79.9 (17.3–99.7)		30.6 (7.6)	29.9 (10.7–51.9)	
Positive	75.9 (13.7)	79.0 (17.9–99.1)	0.59	30.8 (7.4)	30.5 (12.4–58.7)	0.65
HER2 status						
Negative	75.6 (14.9)	78.7 (17.3–99.1)		30.6 (7.6)	30.2 (10.7–58.7)	
Positive	79.1 (11.2)	80.9 (30.7–99.7)	0.36	30.6 (7.4)	30.7 (14.1–49.7)	0.75
Grade						
Low	82.9 (11.0)	84.0 (35.9–98.9)		26.2 (6.9)	26.8 (12.4–38.9)	
Intermediate	80.6 (10.0)	82.4 (35.8–99.7)		30.4 (7.7)	30.5 (11.2–47.6)	
High	73.1 (15.8)	76.4 (13.9–99.1)	<0.0001	32.0 (7.5)	31.9 (10.7–58.7)	<0.0001
Size						
< 2cm	79.0 (14.7)	83.4 (31.5–94.3)		30.4 (8.7)	29.9 (13.1–51.9)	
2–5cm	75.4 (15.4)	78.2 (14.0–99.3)		30.1 (7.5)	30.0 (10.7–58.7)	
>5cm	76.5 (13.9)	79.1 (17.9–99.7)	0.57	31.5 (7.5)	31.2 (11.2–54.3)	0.12
Subtype						
Luminal A-like	77.2 (12.9)	80.6 (17.9–98.9)		30.8 (7.6)	30.3 (12.4–58.7)	
Luminal B-like	76.3 (13.8)	78.6 (30.8–99.1)		30.1 (7.5)	29.8 (10.7–51.9)	
HER2-enriched	80.8 (10.8)	82.4 (51.7–99.7)		32.1 (7.7)	31.9 (17.5–49.7)	
TNBC	74.5 (16.1)	77.7 (17.3–99.1)	0.12	30.9 (7.3)	30.4 (12.1–49.7)	0.75
Age at Menarche, years						
<15	75.1 (14.6)	76.7 (17.8–99.1)		31.5 (7.4)	31.3 (13.6–51.9)	
15	77.4 (14.1)	80.8 (17.3–98.8)		30.7 (7.7)	30.5 (10.7–52.5)	
16	75.4 (15.4)	79.2 (13.9–95.1)		31.0 (6.8)	30.6 (14.4–48.8)	
≥17	77.0 (13.9)	79.9 (18.3–99.1)	0.13	29.7 (7.8)	29.1 (11.2–54.3)	0.12
Parity						
Nulliparous	75.5 (13.8)	78.4 (19.3–96.7)		28.9 (7.1)	28.7 (13.1–46.8)	
Parous	76.2 (14.5)	79.0 (13.9–99.7)	0.71	31.3 (7.6)	31.0 (10.7–58.7)	0.01
Breastfeeding, months						
<13	75.9 (14.3)	78.6 (17.3–99.3)		30.2 (7.4)	29.9 (13.0–52.5)	
13–18	76.8 (13.6)	79.3 (18.3–99.1)		31.4 (7.6)	31.0 (10.7–58.7)	
≥19	74.4 (17.6)	78.6 (13.9–98.9)	0.80	31.3 (8.0)	31.3 (12.4–49.7)	0.34
Body Size						
Slight	76.0 (12.3)	78.4 (30.7–98.1)		31.6 (7.4)	32.0 (12.1–54.3)	
Moderate	75.6 (15.9)	79.0 (17.3–99.3)		31.4 (7.3)	31.3 (13.1–49.7)	
Heavy	77.0 (14.2)	80.6 (13.9–99.1)	0.27	30.1 (8.0)	29.0 (10.7–58.7)	0.005
Family history						
None	76.2 (14.4)	78.7 (13.9–99.6)		30.9 (7.6)	30.5 (11.2–58.7)	
Yes	76.6 (15.1)	81.3 (17.3–99.1)	0.32	33.0 (7.5)	33.4 (10.7–48.2)	0.01

* P values were based on Kruskal Wallis test. ER, estrogen receptor; PR, progesterone receptor; HER2, human epidermal growth factor receptor 2; TNBC: Triple Negative Breast Cancer

**Table 3:** Beta (β) coefficients and 95% confidence intervals (CI) for the associations between patients’ clinicopathological characteristics and tumor microenvironment phenotypes (i.e., Tumor-Stroma Ratio (TSR) and Tumor-associated Stromal Cellular Density (Ta-SCD)) among patients participating in the Ghana Breast Health Study

	Tumor-Stroma Ratio (TSR)				Tumor-associated Stromal Cellular Density (SCD)			
	Partially-adjusted		Multivariable		Partially-adjusted		Multivariable	
Characteristic	β (95% CI)	p-trend	β (95% CI)	p-trend	β (95% CI)	p-trend	β (95% CI)	p-trend
Age (years)								
<35	1.00 (reference)		1.00 (reference)		1.00 (reference)		1.00 (reference)	
35–44	−1.38 (−5.05, 2.29)		−1.60 (−5.19, 1.98)		−0.94 (−2.88, 1.00)		−0.69 (−2.64, 1.25)	
45–54	−1.59 (−5.19, 2.01)		−2.58 (−6.08, 0.93)		−1.42 (−3.33, 0.47)		−0.80 (−2.71, 1.11)	
≥55	−1.06 (−4.57, 2.45)	0.76	−1.44 (−4.85, 1.97)	0.62	0.04 (−1.81, 1.90)	0.51	0.33 (−1.52, 2.19)	0.31
ER status								
Negative	1.00 (reference)		1.00 (reference)		1.00 (reference)		1.00 (reference)	
Positive	3.20 (0.82, 5.57)	0.009	2.91 (0.19, 5.63)	0.03	−1.10 (−2.30, 0.11)	0.07	−0.60 (−2.39, 1.19)	0.49
PR status								
Negative	1.00 (reference)		1.00 (reference)		1.00 (reference)		1.00 (reference)	
Positive	0.13 (−3.04, 3.28)	0.93	−2.05 (−6.08, 0.93)	0.19	−0.18 (−1.65, 1.28)	0.80	0.38 (−1.60, 2.36)	0.69
HER2 status								
Negative	1.00 (reference)		1.00 (reference)		1.00 (reference)		1.00 (reference)	
Positive	3.58 (0.67, 6.49)	0.010	4.39 (1.43, 7.35)	0.005	0.26 (−1.30, 1.81)	0.74	−0.03 (−1.65, 1.60)	0.97
Subtype								
Luminal A	1.00 (reference)		1.00 (reference)		1.00 (reference)		1.00 (reference)	
Luminal B	−1.34 (−4.34, 1.65)	0.38	−0.60 (−3.54, 2.35)	0.69	−0.65 (−2.24, 0.94)	0.42	−0.78 (−2.37, 0.80)	0.33
HER2-enriched	2.59 (−2.07, 7.25)	0.28	3.88 (−0.67, 8.44)	0.09	1.39 (−1.09, 3.87)	0.27	0.99 (−1.46, 3.44)	0.43
TNBC	−4.33 (−7.58, −1.09)	0.009	−2.50 (−5.74, 0.73)	0.13	0.37 (−1.35, 2.61)	0.67	−0.36 (−2.10, 1.36)	0.68
Grade								
Low	1.00 (reference)		1.00 (reference)		1.00 (reference)		1.00 (reference)	
Intermediate	−2.15 (−6.60, 2.30)		−2.07 (−6.70, 2.56)		3.69 (1.21, 6.18)		3.53 (1.03, 6.03)	
High	−9.41 (−13.48, −5.34)	<0.0001	−9.19 (−13.40, −4.98)	<0.0001	5.48 (3.09, 7.87)	<0.0001	5.22 (2.74, 7.71)	<0.0001
Size								
< 2cm	1.00 (reference)		1.00 (reference)		1.00 (reference)		1.00 (reference)	
2–5cm	−2.32 (−8.66, 4.02)		−1.96 (−7.95, 4.03)		−0.01 (−3.19, 3.18)		0.07 (−3.10, 3.23)	
>5cm	−1.00 (−7.04, 5.04)	0.45	−0.93 (−6.69, 4.83)	0.52	1.12 (−2.02, 4.26)	0.07	1.20 (−1.95, 4.36)	0.06

Partially-adjusted linear regression models were adjusted for the relevant clinicopathological characteristic in addition to study site and tissue area. Multivariable linear regression models were mutually adjusted for all clinicopathological characteristics in addition to study site and tissue area.

**Table 4:** Beta (β) coefficients and 95% confidence intervals (CI) for the associations between breast cancer risk factors and tumor microenvironment phenotypes (i.e., Tumor-Stroma Ratio (TSR) and Tumor-associated Stromal Cellular Density (Ta-SCD)) among patients participating in the Ghana Breast Health Study

	Tumor-Stroma Ratio (TSR)				Tumor-associated Stromal Cellular Density (SCD)			
	Univariable		Multivariable		Univariable		Multivariable	
Characteristic	β (95% CI)	p-trend	β (95% CI)	p-trend	β (95% CI)	p-trend	P (95% CI)	p-trend
Age at Menarche, years								
<15	1.00 (reference)		1.00 (reference)		1.00 (reference)		1.00 (reference)	
15	2.63 (−0.33, 5.58)		2.51 (−0.53, 5.55)		−0.75 (−2.31, 0.80)		−0.94 (−2.51, 0.62)	
16	0.72 (−2.38, 3.81)		0.52 (−2.65, 3.69)		−0.50 (−2.13, 1.14)		−0.39 (−2.02, 1.23)	
≥17	1.84 (−1.17, 4.85)	0.42	1.23 (−1.93, 4.39)	0.74	−1.75 (−3.34, −0.17)	0.04	−1.78 (−3.36, −0.19)	0.05
Parity								
Nulliparous	1.00 (reference)		1.00 (reference)		1.00 (reference)		1.00 (reference)	
Parous	0.80 (−2.71, 4.32)	0.65	1.08 (−2.55, 4.70)	0.56	2.45 (0.60, 4.31)	0.01	2.92 (1.04, 4.81)	0.002
Number of children								
1	1.00 (reference)		1.00 (reference)		1.00 (reference)		1.00 (reference)	
2	−1.08 (−4.55, 2.39)		−1.10 (−4.63, 2.43)		0.71 (−1.37, 2.80)		0.75 (−1.36, 2.87)	
3	0.08 (−3.34, 3.51)		0.05 (−3.42, 3.53)		−0.90 (−2.96, 1.16)		−0.84 (−2.93, 1.24)	
4	−0.14 (−3.62, 3.34)		−0.001 (−3.54, 3.54)		−0.03 (−2.12, 2.06)		−0.12 (−2.24, 2.00)	
≥5	−1.70 (−4.80, 1.39)	0.32	−1.26 (−4.50, 1.97)	0.58	0.56 (−1.30, 2.42)	0.63	0.57 (−1.37, 2.51)	0.73
Age at first birth (years)								
<19	1.00 (reference)		1.00 (reference)		1.00 (reference)		1.00 (reference)	
19–21	0.66 (−2.06, 3.38)		0.63 (−2.19, 3.45)		1.39 (−0.05, 2.83)		1.80 (0.34, 3.26)	
22–25	2.50 (−0.31, 5.31)		2.30 (−0.61, 5.22)		−0.37 (−1.86, 1.12)		0.05 (−1.46, 1.57)	
≥26	1.78 (−1.26, 4.82)		1.78 (−1.31, 4.87)	0.11	0.88 (−0.72, 2.50)	0.73	1.28 (−0.32, 2.89)	0.39
Breastfeeding, months								
<13	1.00 (reference)		1.00 (reference)		1.00 (reference)		1.00 (reference)	
13–18	0.60 (−1.99, 3.20)		0.73 (−1.94, 3.40)		0.61 (−0.76, 1.98)		0.75 (−0.63, 2.13)	
≥19	−2.08 (−5.72, 1.55)	0.41	−2.12 (−5.95, 1.71)	0.45	0.69 (−1.23, 2.61)	0.41	0.53 (−1.46, 2.52)	0.47
Parity/b reastfeed i n g (months)								
Nulliparous	1.00 (reference)		1.00 (reference)		1.00 (reference)		1.00 (reference)	
Parous/<13	0.89 (−3.12, 4.89)	0.66	0.78 (−3.28, 4.83)	0.71	2.09 (−0.02, 4.21)	0.05	2.48 (0.37, 4.61)	0.02
Parous/13–18	1.50 (−2.18, 5.18)	0.42	1.63 (−2.11, 5.38)	0.39	2.76 (0.82, 4.70)	0.005	3.34 (1.39, 5.30)	0.001
Parous/≥19	−1.24 (−5.81, 3.32)	0.59	−1.27 (−5.91, 3.37)	0.59	2.73 (0.33, 5.13)	0.02	3.36 (0.93, 5.78)	0.007
Body Size								
Slight	1.00 (reference)		1.00 (reference)		1.00 (reference)		1.00 (reference)	
Moderate	−0.74 (−3.40, 1.93)		−0.68 (−3.42, 2.05)		−0.41 (−1.80, 0.98)		−0.68 (−2.07, 0.72)	
Heavy	0.52 (−2.22, 3.28)	0.64	0.48 (−2.36, 3.33)	0.67	−1.82 (−3.22, −0.42)	0.008	−2.14 (−3.56, −0.72)	0.002
Family history								
None	1.00 (reference)		1.00 (reference)		1.00 (reference)		1.00 (reference)	
Yes	0.62 (−3.33, 4.57)	0.76	0.89 (−3.10, 4.87)	0.66	2.31 (0.22, 4.40)	0.03	2.36 (0.28, 4.44)	0.02

Partially-adjusted linear regression models were adjusted for the relevant risk factor in addition to age, study site, and tissue area. The primary multivariable linear regression model was mutually adjusted for age at menarche, parity, body size, family history of breast cancer in a first degree relative, age, study site and tissue area. In secondary models, parity was substituted for number of children among parous women, or age at first birth, or breastfeeding, or parity/breastfeeding.

## Data Availability

All the datasets used and/or analyzed during the current study are available from the corresponding author on reasonable request.

## Abbreviations

BMI: Body mass index
CI: Confidence interval
ECM: Extracellular matrix
ER: Estrogen receptor
FHBC: Family history of breast cancer
GBHS: Ghana breast health study
HER2: Human epidermal growth factor receptor 2
IHC: Immunohistochemical
MHT: Menopausal hormone therapy
OR: Odds ratio
PR: Progesterone receptor
SME: Stromal microenvironment
Ta-SCD: Tumor-associated stromal cellular density
TNBC: Triple negative breast cancer
TSR: Tumor-stroma ratio
